# The digestive system and autoimmunity

**DOI:** 10.1186/s12865-023-00561-4

**Published:** 2023-10-04

**Authors:** Lina Sun, Baojun Zhang

**Affiliations:** 1 Department of Pathogenic Microbiology and Immunology, School of Basic Medical Sciences, Xi’an Jiaotong University, 710061 Xi’an, Shaanxi China; 2 Institute of Infection and Immunity, Xi’an Jiaotong University Health Science Center, Translational Medicine Institute, 710061 Xi’an, Shaanxi China; 3 Key Laboratory of Environment and Genes Related to Diseases, Ministry of Education, 710061 Xi’an, Shaanxi China; 4 Xi’an Key Laboratory of Immune Related Diseases, 710061 Xi’an, Shannxi China

## Abstract

Digestive autoimmune conditions are a growing challenge to global health. Risk factors associated with autoimmune digestive diseases are complex, including genetic variation, immunological dysfunction, and various environmental factors. To improve our understanding of the mechanisms behind digestive autoimmune conditions, including factors causing gastrointestinal manifestations and pathogenesis, *BMC Immunology* has launched a new Collection “The digestive system and autoimmunity”.

Failure of immune tolerance causes self-attacking and autoimmune disorders. To date, more than 80 types of autoimmune diseases have been characterized, affecting approximately 5–8% of the world’s population. In recent years, both the incidence and prevalence of autoimmune diseases has increased rapidly. Though medical interventions can mitigate the symptoms, autoimmune diseases cannot be cured. Autoimmune diseases can be systemic or at local sites, including the gastrointestinal tract. Autoimmune disorders associated with the digestive system, lead to a variety of gastrointestinal manifestations, such as oral ulcers, abdominal pain, diarrhea, and gastrointestinal bleeding [1]. Moreover, evidence has suggested that persistent autoimmune inflammation and immunosuppressive therapy have been linked to increased cardiovascular risk or cancer [2].

The mechanisms behind gastrointestinal autoimmune diseases are complex with various causal factors, including genetic variation, immunological dysfunction, environmental factors, infections, diet and gut microbiota. Advances in next-generation sequencing technology, such as genome-wide association studies (GWAS), have revealed hundreds of genomic variants that highly influence the risk of autoimmune diseases [3]. A balanced immune system is important for maintaining homeostasis by protecting the host from infection and preventing self-reactivity. Dysfunction of immune homeostasis causes inflammation, a key immunological determinant of autoimmune disorders. Abnormal inflammation, which causes substantial tissue damage, is strongly linked to autoimmune pathogenesis.

T cell-mediated inflammatory responses are important drivers for immunopathogenesis in many autoimmune disorders. Pro-inflammatory CD4^+^ T helper (Th) cells play critical roles in promoting the pathogenesis and progression of autoimmune diseases [4]. Pro-inflammatory cytokines produced by those cells are potent activators of other immune cells (e.g., neutrophils and macrophages) and non-immune cells (e.g., epithelial, endothelial cells and fibroblasts) to amplify the inflammatory response. Follicular helper T (Tfh) cells are also highly associated with a wide range of autoimmune diseases through their ability to enhance autoreactive B cells and CD8^+^ T cell responses. Further, dysfunction of regulatory T (T_reg_) cells which are the predominant T cell population maintaining self-tolerance, has been linked to most autoimmune manifestations [5].

Emerging evidence has revealed that environmental stimulators, such as chemical toxins, solvents, organic pollutants, and heavy metals, are additional risk factors driving digestive autoimmunity. Several underlying mechanisms have been identified linking toxic chemicals to immune dysregulation [6]. Notably, various infectious agents can trigger autoimmune diseases in the gastrointestinal tract. Multiple mechanisms by which pathogenic infections cause autoimmune disorders have been characterized, such as molecular mimicry, epitope spreading, bystander activation, and induction of inflammatory environment [6].

The gastrointestinal tract is now considered the largest immunological organ since it harbors 70% of the body’s lymphocyte population [7]. Given that the gastrointestinal tract is home to multitudes of commensal bacteria, it is now well-appreciated that microbiota play pivotal roles in shaping the development of the immune system and regulating immune responses. In recent years, microbiota have also strongly correlated with digestive autoimmune disease [8]., The phyla of gut microbiota in IBD patients significantly differ from that in healthy individuals. This dysbiosis—an abnormal microbiome immunity— affects both the innate and adaptive immune systems, promoting pro-inflammatory responses and autoimmunity [8]. Moreover, gut microbiota can influence extraintestinal autoimmune disorders, such as rheumatoid arthritis (RA), type 1 diabetes (T1D) and experimental autoimmune encephalomyelitis (EAE) [8]. Therefore, any practice that alters the composition of gut microbiota, such as dietary habit, physical activity, antibiotic or probiotics treatments and vaccinations, can affect digestive autoimmune diseases. It has been implicated that a high; fat, salt and sugar diet has a strong association with increased pathogenic Th17 cell differentiation and the development of autoimmunity [9]. However, a fiber-rich diet favours the presence of *Faecalibacterium prausnitzii* species, which could protect the host from colonic inflammation [10]. Host–microbiota interactions significantly modulate the mucosal immune responses and contribute to the pathogenesis of autoimmune diseases.

Traditional therapeutic drugs for autoimmune diseases are generally nonspecific immunosuppressants, such as nonsteroid anti-inflammatory drugs (NSAIDs), steroid anti-inflammatory drugs (SAIDs) and disease-modifying antirheumatic drugs (DMARDs). In the past decades, immunological drugs targeting inflammatory cytokines, receptors, intracellular molecules and kinase pathways have been developed and revolutionized the treatment of autoimmune diseases. Novel therapeutic targets are also under evaluation, such as TGFβ-SMAD signalling, cell adhesion or migration molecules and the NLRP3 inflammasome [11]. Various novel immunotherapies engaging in T-cell immunity have recently been applied in autoimmune diseases and demonstrated promising therapeutic efficacy [4]. In reference to immunotherapies in cancer, engineered chimeric antigen receptor (CAR)-T cells and chimeric autoantibody receptor (CAAR)-T cells are developed to target autoreactive B cells or T cells in autoimmune diseases. In addition, strategies to induce T_reg_ cell expansion and function or generate CAR-T_reg_ cells have been extensively studied in preclinical autoimmune models [12].

In conclusion, the prevalence of autoimmune diseases of the digestive system is rising rapidly worldwide. A better understanding of the cellular and molecular signalling pathways and regulatory mechanisms offers valuable insights into developing novel prophylactic and therapeutic strategies for autoimmune diseases. Hence, we are now calling for submissions to our Collection on “The digestive system and autoimmunity” to highlight this issue. We hope this Collection will attract people’s attention to autoimmune digestive diseases and inspire researchers to continually investigate the underlying mechanism and develop effective therapeutic strategies.

## Data Availability

Not applicable.
